# Supplementary material for: MetaQTL: a package of new computational methods for the meta-analysis of QTL mapping experiments
Source: BMC Bioinformatics. 2007 Feb 8;8:49. doi: 10.1186/1471-2105-8-49 (PMC1808479; doi:10.1186/1471-2105-8-49)
Supplement: Additional File 7 — MetaQTL Package : jar file and tutorial. This Zip archive contains both the MetaQTL JAR file and the files of the tutorial. [file 1471-2105-8-49-S7.zip › org.inra.metaqtl/doc/org/metaqtl/bio/entity/GeneticMap.html]

GeneticMap


|  |  |  |
| --- | --- | --- |
| **Overview** \| **Package** \| **Class** \| **Use** \| **Tree** \| **Deprecated** \| **Index** \| **Help** | |  |
| **PREV CLASS**   **NEXT CLASS** | **FRAMES**    **NO FRAMES**     **All Classes** |  |
| SUMMARY: NESTED \| FIELD \| CONSTR \| METHOD | DETAIL: FIELD \| CONSTR \| METHOD |  |


---


## org.metaqtl.bio.entity Class GeneticMap

```
java.lang.Object
  org.metaqtl.bio.entity.BioEntity
      org.metaqtl.bio.entity.BioEntityContainer
          org.metaqtl.bio.entity.GroupContainer
              org.metaqtl.bio.entity.GeneticMap
```

**All Implemented Interfaces:**: IBioAdaptable, IBioEntity, IBioGenome

---

``` public class GeneticMap extends GroupContainer ```

Class Description Here

**Author:**
:   Jean-Baptiste Veyrieras

---

**Field Summary**

**Fields inherited from class org.metaqtl.bio.entity.BioEntityContainer**

`entities`

**Fields inherited from class org.metaqtl.bio.entity.BioEntity**

`name, parent, properties`


**Constructor Summary**

`GeneticMap()`

`GeneticMap(java.lang.String name)`

`GeneticMap(java.lang.String name,
IBioEntity parent)`


| **Method Summary** | |
| --- | --- |
| `IBioAdapter` | `getBioAdapter()` |
| `int` | `getType()`             There are 2 main class of entities. |
| `GeneticMap` | `merge(GeneticMap map)`             **Deprecated.** |

**Methods inherited from class org.metaqtl.bio.entity.GroupContainer**

`addGroup, getGroup, groupNumber, groups, removeGroup`

**Methods inherited from class org.metaqtl.bio.entity.BioEntityContainer**

`addEntity, entities, entityNumber, getEntity, removeEntity`

**Methods inherited from class org.metaqtl.bio.entity.BioEntity**

`getName, getParent, getProperties, newBioEntity, setName, setProperties`

**Methods inherited from class java.lang.Object**

`clone, equals, finalize, getClass, hashCode, notify, notifyAll, toString, wait, wait, wait`

**Methods inherited from interface org.metaqtl.bio.IBioEntity**

`getName, getParent, getProperties, setName, setProperties`

**Constructor Detail**

### GeneticMap

```
public GeneticMap()
```

---


### GeneticMap

```
public GeneticMap(java.lang.String name,
                  IBioEntity parent)
```

**Parameters:**: `name` -: `parent` -

---


### GeneticMap

```
public GeneticMap(java.lang.String name)
```

**Parameters:**: `name` -


**Method Detail**

### merge

```
public GeneticMap merge(GeneticMap map)
```

:   **Deprecated.** 

    :   **Parameters:**: `map` - **Returns:**

---


### getType

```
public int getType()
```

:   **Description copied from interface: `IBioEntity`**
:   There are 2 main class of entities. The first one deals with population
    biological entity, i.e population itself and individuals. The second class
    is a representation of microscopic biological entity from genome container
    to alleles.

    :   **Specified by:**: `getType` in interface `IBioEntity` **Specified by:**: `getType` in class `GroupContainer`

---


### getBioAdapter

```
public IBioAdapter getBioAdapter()
```


---


|  |  |  |
| --- | --- | --- |
| **Overview** \| **Package** \| **Class** \| **Use** \| **Tree** \| **Deprecated** \| **Index** \| **Help** | |  |
| **PREV CLASS**   **NEXT CLASS** | **FRAMES**    **NO FRAMES**     **All Classes** |  |
| SUMMARY: NESTED \| FIELD \| CONSTR \| METHOD | DETAIL: FIELD \| CONSTR \| METHOD |  |


---
